# Efficient construction and utilization of *k*-ordered FM-indexes with kISS for ultra-fast read mapping in large genomes

**DOI:** 10.1093/bioinformatics/btae409

**Published:** 2024-06-19

**Authors:** Zheng-Dao Yang, Hsuan-Yu Kuo, Po-Wei Hsieh, Jui-Hung Hung

**Affiliations:** Department of Computer Science, National Yang Ming Chiao Tung University, Hsinchu 300093, Taiwan; Department of Computer Science, National Yang Ming Chiao Tung University, Hsinchu 300093, Taiwan; Department of Computer Science, National Yang Ming Chiao Tung University, Hsinchu 300093, Taiwan; Department of Computer Science, National Yang Ming Chiao Tung University, Hsinchu 300093, Taiwan

## Abstract

**Motivation:**

The Full-text index in Minute space (FM-index) is a memory-efficient data structure widely used in bioinformatics for solving the fundamental pattern-matching task of searching for short patterns within a long reference. With the demand for short query patterns, the *k*-ordered concept has been proposed for FM-indexes. However, few construction algorithms in the state of the art fully exploit this idea to achieve significant speedups in the pan-genome era.

**Results:**

We introduce the *k*-ordered induced suffix sorting (kISS) for efficient construction and utilization of *k*-ordered FM-indexes. We present an algorithmic workflow for building *k*-ordered suffix arrays, incorporating two novel strategies to improve time and memory efficiency. We also demonstrate the compatibility of integrating *k*-ordered FM-indexes with locate operations in FMtree. Experiments show that kISS can improve the construction time, and the generated *k*-ordered suffix array can also be applied to FMtree without any additional in computation or memory usage.

**Availability and implementation:**

https://github.com/jhhung/kISS.

## 1 Introduction

Read mapping is a fundamental process in bioinformatics used to determine the positions of short DNA or RNA sequences (i.e. reads) within a longer reference genome. The goal of read mapping is to find the optimal alignment or mapping location for each read within the reference genome. The process typically begins with seeding, which involves identifying short, exact, or nearly exact matches (i.e. seeds) between a read and the reference genome. A subset of seeds, known as seed chains, which are well-distributed along both the read and the reference genome, is then used to construct the final alignment, considering gaps, mismatches, and insertions or deletions (indels) (Li 2013, 2018, Kim *et al.* 2019, Chen *et al.* 2021).

Efficient seeding is achieved by indexing the occurrences and coordinates of seeds in the reference sequence using two strategies: hashing and the FM-index. Hashing is known for its ease of implementation and quick query times but tends to consume more space and can only index fixed-size seeds. In contrast, the FM-index is space-efficient and capable of indexing variable-length seeds but requires more complex implementation and has slower querying performance. Although contemporary computer memory capacities have substantially increased, making hash indexing increasingly popular (Alser *et al.* 2021), the pan-genome era is marked by a growing number of available reference genomes (Liao *et al.* 2023) and the need to process batch samples in parallel. This shift underscores a pressing demand for more memory- and computation-efficient seed indexing methods. This work addresses two primary drawbacks of the FM-index—its implementation complexity and slower query time—with the goal of enhancing seed indexing for large genomes.

The FM-index, when built on a reference sequence in advance, offers pattern-searching functionality. It supports two primary functions: count and locate. The count operation retrieves the number of occurrences of a query string, while the locate operation identifies the specific positions of these occurrences in the reference sequence. The efficiency of FM-indexes relies heavily on the efficiency of their construction and the implementation of these functions.

The crucial component in constructing the FM-index is the Burrows-Wheeler Transform (BWT) (Ferragina and Manzini 2000) of the reference sequence, derived from the suffix array as part of its construction process (see Supplementary Materials S1 and S2 for details). Various algorithms have been proposed for efficiently constructing suffix arrays (Puglisi *et al.* 2007). Among these, induced-sorting-based algorithms (Burrows 1994) have shown superior linear time complexity in practical applications. The key insight of induced-sorting-based algorithms is that a complete sort of the selected suffixes can be used to induce a complete sort of the other suffixes. The most well-known algorithm using this technique is the SA-IS algorithm (Nong *et al.* 2009), which induces the complete sort from the leftmost S-type suffixes (LMS suffixes for short; see Supplementary Materials S1 for details).

Numerous techniques have been applied to improve the performance of the SA-IS algorithm. Notably, SACAK (Nong 2013) stands out as the most space-efficient implementation of SA-IS, requiring only O(1) extra workspace for constant alphabets. Conversely, pSAIS (Lao *et al.* 2018a,b) represents a parallelized version of the SA-IS algorithm, showcasing a high degree of parallelism. Recent developments include pSACAK (Lao *et al.* 2018a,b) and pSACAK+ (Xie *et al.* 2020), introducing parallel variants of SACAK that achieve remarkable efficiency in terms of both time and space. On a different note, parDSS (or pDSS) (Labeit *et al.* 2017), also rooted in the induced-sorting concept, takes slightly longer when dealing with references containing numerous repeats. However, it demonstrates commendable performance with most real-world references, and some experiments even reveal that parDSS outperforms pSACAK+ in certain scenarios (Xie *et al.* 2020).

Unfortunately, none of these implementations can be characterized as straightforward. Their complexity is necessary to handle the ubiquitous long repeats present in large genomes. However, modern read mapping algorithms often require only short seeds, typically <32 base pairs. The complexity can be significantly reduced if these repetitions can be made insignificant. Chang *et al.* (2016) have shown that a *k*-ordered FM-index based on the Schindler transform (Schindler 1997, Ge and Sen 2006), where the order of the suffixes is solely decided by their first *k*-mers, can adequately serve the purpose of seeding in read mapping without addressing the repeats. The proposed sBWT algorithm can achieve simplicity in implementation, but construction remains time-intensive and the enumeration step essential for the search phase introduces computational overhead to the algorithm.

Regarding the implementation of the count and locate operations, in an effort to reduce space usage, two types of sampling strategies, subscript sampling and value sampling, have been applied to the constructed data structures (see Supplementary Material S2 for details). While these strategies reduce space consumption, they also slow down the locate operations. Recently, FMtree (Cheng *et al.* 2018) has provided an effective solution to this problem. FMtree achieves a 10-fold speedup compared to other implementations, resulting from the reduction of unnecessary operations in the locate function by structuring the search space as a conceptual multiway tree.

In light of these advances and challenges, we propose efficient induced-sorting-based algorithms for the simple construction of *k*-ordered FM-indexes. We demonstrate their easier implementation, superior time efficiency, and moderate memory consumption on real genomic data. In addition, we show that *k*-ordered FM-indexes can further benefit from FMtree for the locate function. By integrating these components, we introduce kISS as a cohesive solution, overcoming the drawbacks of traditional FM-index-based seed indexing methods, ready to seamlessly integrate into the existing pipeline for read mapping in the pan-genome era.

## 2 Materials and methods

In this section, we describe how kISS constructs a *k*-ordered FM-index using the induced-sorting-based algorithm (see Section 2.1). We also demonstrate how the *k*-ordered FM-index, when integrated with FMtree (Cheng *et al.* 2018), avoids the enumeration step crucial for the search phase—a step typically required by sBWT (Chang *et al.* 2016)—without additional computational or memory demands (see Section 2.2).

### 2.1 The induced-sorting-based *k*-ordered FM-index construction algorithms

**Figure 1.**
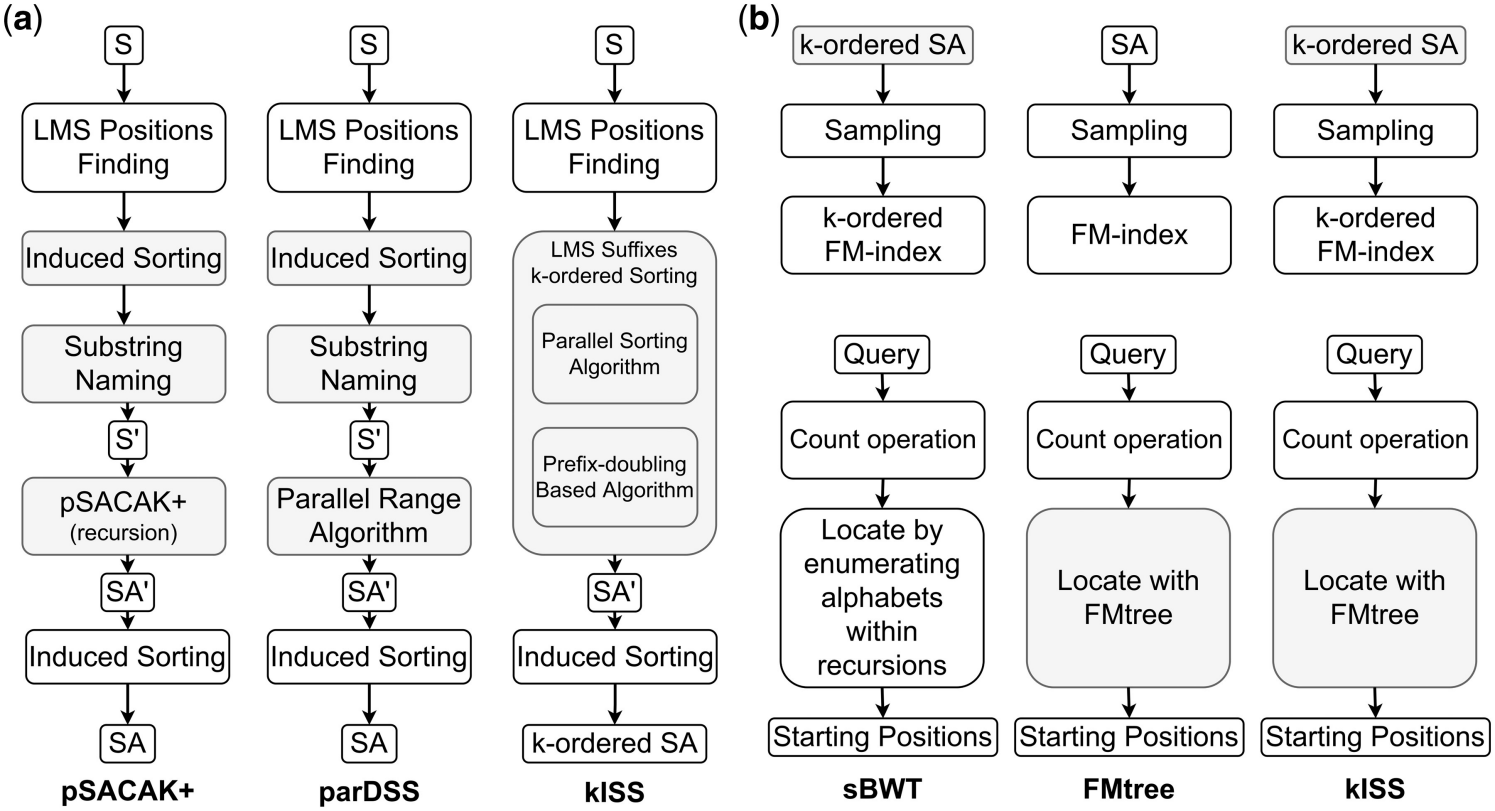
Highlights of the kISS algorithm. (a) The *k*-ordered Suffix Array Construction Algorithm designed for *k*-ordered FM-indexes, compared to the methods used by pSACAK+ and parDSS. All three algorithms are based on induced sorting, which determines the complete order of suffixes starting from the LMS suffixes. They begin by identifying LMS positions in S (the input reference) and sort the LMS suffixes differently. Both pSACAK+ and parDSS focus on LMS substrings, going through stages like executing Induced Sorting (sorting all LMS substrings) and performing Substring Naming (renaming each LMS substring by their rank to create a shortened string S′). However, while pSACAK+ solves the subproblem recursively, parDSS uses the Parallel Range algorithm, a prefix doubling technique to solve the reduced subproblem. In contrast, kISS directly sorts the LMS suffixes by their initial k-mers. The algorithms conclude with another round of Induced Sorting (deriving the order of all suffixes from the sorted LMS suffixes SA′). (b) The FM-index construction process and the locate operation, compared with the methodologies used by sBWT, FMtree, and kISS. In constructing FM-indexes, both sBWT and kISS utilize *k*-ordered suffix arrays as input, differing from FMtree’s use of conventional suffix arrays. Despite this variation, the methods employed by all three are similar. During locate operations, after the count operation is completed, sBWT requires enumerating all strings from length 1 to *vd* (where *vd* denotes the value sampling distance). FMtree and kISS both utilize an analogous conceptual multiway tree structure to facilitate locate operations.

A *k*-ordered FM-index resembles the FM-index, but replaces the BWT by the Schindler transform (ST). The ST, a variant of the BWT, arranges circular shifts of the reference solely based on prefixes of length *k* (Schindler 1997, Ge and Sen 2006). Literature on constructing *k*-ordered FM-indexes has demonstrated that building a *k*-ordered suffix array is essential (Fig. 1b; Chang *et al.* 2016). This array ensures that suffixes are sorted only by their first *k*-mers. We first introduce the *k*-ordered induced-sorting-based suffix array construction algorithm, utilizing it as a subroutine of the ST.

The core principle behind developing this algorithm is to incorporate state-of-the-art induced-sorting-based suffix array construction, leveraging its superior time and space complexities within the framework of the simplistic *k*-ordered sorting scheme. kISS adopts a framework similar to pSACAK+ and parDSS. Initially, it categorizes each suffix as L-type or S-type and identifies a selected subset of all suffixes termed LMS suffixes. Subsequently, it sorts the LMS suffixes in lexicographical order and reconstructs the complete order of the suffixes based on that of the LMS suffixes (see Supplementary Material S1 for details).

The main difference lies in how the LMS suffixes are sorted. In pSACAK+ and other SA-IS-based algorithms, a recursive framework is utilized. The framework first derives a compact form of the reference through the step called substring naming. The compact form is designed so that when the suffix array of this compact form is correctly calculated, it is sufficient to determine the correct order of the LMS suffixes. To enable substring naming, another induced sort is necessary (Fig. 1a, left panel; see Supplementary Material S1 for details). Conversely, while parDSS also derives a compacted reference through substring naming, it does not recursively apply parDSS to the subproblem. Instead, the compacted reference undergoes a step called parallel range from the PBBS library (Shun *et al.* 2012), which falls into the prefix-doubling category of suffix array construction algorithms (Fig. 1a, middle panel; see Supplementary Material S1 for more details). The framework of sorting the selected suffixes directly and inducing the order of all suffixes is termed as induced copying (Burrows 1994, Puglisi *et al.* 2007).

The primary limitation of these existing methods is their challenging parallelization due to high data dependency. Both algorithms rely on induced sorting followed by substring naming, which serves as a major performance bottleneck. SA-IS-based algorithms break down into subproblems, further impeding parallelization across iterations and limiting achievable parallelism. In contrast, kISS takes a different approach by sorting the LMS suffixes based on their first *k*-mers, this avoiding the need for a compacted representation through substring naming (Fig. 1a, right panel). Crucially, to surpass the performance of pSACAK+ or parDSS, it is essential to use methods that facilitate parallelism. Here, we present two strategies for sorting the LMS suffixes based solely on their positions: the parallelized string sorting algorithm (see Section 2.1.1) and the prefix-doubling-based algorithm (see Section 2.1.2).

#### 2.1.1 Strategy 1: parallelized string sorting algorithm

**Figure 2.**
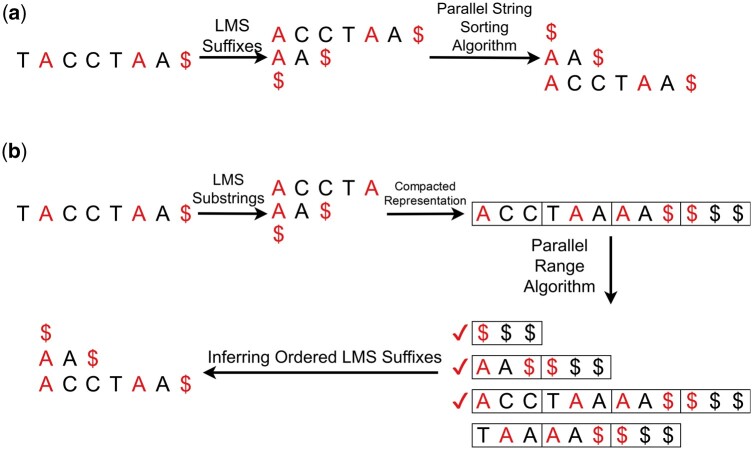
The two strategies for performing k-ordered sorting of LMS suffixes. (a) The first strategy finds out all the LMS suffixes (namely, ACCTAA$, AA$, $ in this example) and performs a parallelized sort. (b) The second strategy initially encodes each LMS substring into a smaller string. Consecutive l-mers (e.g. l = 3) are encoded into a single integer (represented by a box). For example, taking the first LMS substring ACCTA, ACC, and TAA are considered as two new characters. Since the length is not divisible by three, the last segment, TAA, includes prefixes from the subsequent LMS substring. Subsequently, prefix doubling is performed on the encoded string to obtain the correct order of the LMS suffixes.

This strategy directly sorts LMS suffixes using well-established parallel sorting algorithms, which efficiently handle only the initial k-mers of each suffix (Bingmann *et al.* 2017) (see Fig. 2a**)**. By focusing solely on these *k*-mers, the direct application of these algorithms to LMS suffixes proves to be both effective and efficient.

For simplicity, we implemented kISS using a parallel multikey sort algorithm, the default mechanism employed by the std::sort function within the C++ standard library. We enhanced the performance of std::sort by incorporating the std::execution::parallel_policy, which allows for the parallelization of the algorithm’s execution for optimized efficiency. To further optimize our algorithm, we included Single Instruction-Multiple Data (SIMD) instructions. Utilizing x86 Intrinsics for steps like fetching *k*-mers and comparing them can significantly speed up the process (see Supplementary Material S3 for details).

Inspired by the split sort from sBWT, we also implemented a variant of this method. This variant places the LMS suffixes into buckets based on their first *l*-mers (e.g., *l *=* *10), and then sorts the suffixes within each bucket. In our experiments, this modification provided an additional 1.5× speedup compared to the original implementation.

#### 2.1.2 Strategy 2: prefix-doubling-based algorithm

The primary concern with Strategy 1 (kISS-1) is its inferior worst-case time complexity, which results in drastically increased time usage when there is an extremely high repetition in the prefixes of suffixes. This issue becomes particularly evident in scenarios where a long genome is concatenated with itself. In such cases, the algorithm faces increased time usage due to numerous suffixes sharing identical prefixes. To address this issue, we propose a prefix-doubling-based algorithm. The core concept of this approach involves doubling the length of the prefix of all suffixes used for sorting in each round of a partial sort. This doubling strategy allows for efficiently reaching the correct suffix array by progressively extending the sorting basis, doubling log_2_(*n*) times, where *n* is the length of the reference (Karp *et al.* 1972, Manber and Myers 1993) (see Supplementary Material S1 for details). For example, the parallel range algorithm from PBBS is a parallelized implementation of this algorithm.

We choose the prefix-doubling-based algorithm for several reasons. Firstly, its time complexity is more tightly bounded than that of parallel string sort [O(*n′* log *n′*) compared to O(*n′ k* log *n′*) in the worst case, where *n′* is the number of LMS suffixes]. This results in a more stable runtime, especially when handling larger values of *k* or references with a high number of repeats. Secondly, most prefix-doubling implementations use a few rounds of radix sorts, which are easier to parallelize compared to induced sort or substring naming. Although radix-sorting increases the likelihood of cache misses and remains a bottleneck, this impact can be mitigated by sorting only the LMS suffixes rather than all suffixes. Finally, the algorithm can be easily adapted to a *k*-ordered version by performing doubling only log_2_(*k*) times, enabling prefix-doubling-based algorithms to adopt *k*-ordered sorting schemes.

The main challenge in developing prefix-doubling-based algorithms is that we only have the positions of the LMS suffixes, rather than a compacted representation of the reference. This poses a challenge because doubling heavily relies on the rank of each suffix in the partial order, which cannot be calculated for LMS suffixes without considering other suffixes. To address this issue, we introduce a more parallelism-friendly representation of the reference. This representation reduces the input reference into the concatenation of several integer arrays, where each array represents the encoded LMS substring (i.e. the substring formed by two consecutive occurrences of positions from LMS suffixes). For each LMS substring, the array is formed by dividing it into *l*-mers and encoding each *l*-mer into one integer, where *l* is a predefined integer. The encoding of each *l*-mer is performed by treating it as a base-|*Σ*| number (|*Σ*| representing the alphabet size). In cases where the last substring contains fewer than *l*-mers, prefixes of the subsequent LMS substrings are included. The concatenation of the encoded LMS substrings forms the reduced reference, and additional information, such as the locations of LMS suffixes in the reduced reference, is also computed (see Supplementary Material S4 for the correctness of this encoding step).

This encoding of the reference intuitively reduces the number of comparisons between two suffixes. In most real test cases, especially with small character sets like DNA, the reduced reference is only slightly longer than the number of LMS suffixes (see Supplementary Material S7 for experimental results). This observation suggests that Strategy 2 can be viewed as a simplified version of parDSS. Here, we utilize a slightly longer but computationally simpler representation of the input reference, which avoids the complexity of substring naming. Given that parDSS can outperform pSACAK+ under certain conditions, this strategy may offer comparable advantages. The reduced reference serves as the input for our implementation of the parallel range algorithm. After calculating the suffix array of the reduced reference, the order of LMS suffixes is inferred from the additional information (see Supplementary Material S3 for implementation details).

Users can choose one of two strategies to rank the LMS suffixes. Following this choice, the *k*-ordered suffix array is calculated. Subsequently, the Schindler transform can be performed to construct the *k*-ordered FM-index from the *k*-ordered suffix array.

### 2.2 Applying the locate function in FMtree with *k*-ordered FM-indexes

The *k*-ordered FM-Index, proposed by sBWT (Chang *et al.* 2016), leverages the advantage of *k*-ordered suffix arrays to mitigate the bottleneck encountered in constructing the suffix array, which is also the bottleneck in building the FM-index. Further, the research revealed that even with only *k*-ordered suffix arrays, the FM-index’s count and location operations can still be effectively performed through LF mapping. It imposes only one restriction compared to the traditional FM-Index: the length of the search string cannot exceed *k*.

However, suffix arrays consume significant memory. For instance, for a human genome of length 3G base pairs (bp), the memory space required for its suffix array (SA) is 12G. To reduce memory usage, a common practice in FM-index is suffix array sampling. There are two common methods of suffix array sampling: subscript sampling, which retains elements SA[*i*] where *i* mod *sd *=* *0, and value sampling, which retains elements SA[*i*] where SA[*i*] mod *vd *=* *0 (where *sd* and *vd* are predefined integers indicating the distance for subscript sampling and value sampling respectively).

Despite the benefits of suffix array sampling in reducing memory usage, it introduces challenges for the locate operation of the *k*-ordered FM-Index. The locate algorithm proposed by sBWT considers the set of strings with pattern *s*P, where *s* may be any string of length ranging from 0 to (*vd* - 1), and finds the range in the *k*-ordered suffix array for all strings in the set using the existing count function. However, since it is based on the *k*-ordered sampled suffix array, the process of locating requires enumerating alphabets within recursions. This may result in search overhead and raise doubts about the performance of the *k*-ordered FM-Index when applying suffix array sampling (Fig. 1b, left panel; see Supplementary Material S2 for details).

Faster locate algorithms have been proposed for ordinary value-sampled FM-indexes. The FMtree (Cheng *et al.* 2018) serves as a rapid locate algorithm designed for value-sampled FM-indexes. Its optimization is based on the observation that when the indexes within the suffix array, subjected to LF-mapping operations, are contiguous, numerous LF-mapping operations can be omitted (see Supplementary Material S2 for details). Inspired by this, FMtree organizes the set of strings into a complete |*Σ*|-ary tree (with |*Σ*| representing the alphabet size), where each node contains a string that precedes the string of its parent node by one character. The range for the pattern P can be found with breadth-first search in the tree, and the occurrence positions of P can then be retrieved block-by-block in the sampled suffix array (Fig. 1b, middle panel; see Supplementary Material S2 for details).

In our study, we demonstrate that when the query length does not exceed *k-vd* + 1, the location algorithm in FMtree returns accurate results without any modifications or increases in computational resources, even with *k*-ordered FM-indexes (see Supplementary Material S5 for complete proof; the source code for validating the correctness of kISS with FMtree can be found at https://github.com/JHHLAB/FMtree). Thus, the *k*-ordered suffix array generated by kISS benefits from the efficiency enhancements offered by FMtree. This allows it to be directly utilized as the locate function for the *k*-ordered FM-Index without additional computational or memory overhead (Fig. 1b, right panel).

## 3 Results

The following experiments were conducted on a machine equipped with two Intel Xeon Platinum 8358P CPUs of 32 cores per each and 512 GiB RAM. The operating system used is Ubuntu 22.04 LTS, a 64-bit system. All the programs were compiled using g++ (v11.4.0) with the -Ofast optimization option. Parallelism was achieved by using OpenMP (v4.5.0) and Intel oneAPI Threading Building Blocks (oneTBB) library (v2020.3) for execution policies.

### 3.1 Time and space usage among suffix array construction algorithms

The experimental setup involved evaluating several algorithms under various conditions. The tested algorithms included the following:

- kISS-1: An implementation of kISS that employs Strategy 1 for ordering the LMS suffixes. The experiments tested various *k* values including 2, 4, 8, 16, 32, 64, 128, 256, and *k* = unbounded (representing the conventional suffix array) across different number of threads (1, 2, 4, 8, 16, 32, 64, 128).
- kISS-2: Another implementation of kISS, employing Strategy 2 for ordering the LMS suffixes. The configuration and parameters consistent with those of kISS-1.
- libsais: The pSACAK+ algorithm described in (Xie *et al.* 2020) as the current state-of-the-art and fastest suffix array construction algorithm in most cases. We use the widely accepted implementation from libsais (v2.8.1), available at https://github.com/IlyaGrebnov/libsais.
- SACA-K: Detailed in (Nong 2013), with the code accessible for download from http://code.google.com/p/ge-nong/.
- parDSS: Presented in (Labeit *et al.* 2017), with the code available for download from https://github.com/jlabeit/parallel-divsufsort with the last commit on December 28, 2016.

The runtime of each program was measured using the spdlog library (v1.9.2), while space usage was quantified through the ru_maxrss variable, which records the maximum resident set size using the getrusage POSIX function.

The experimental datasets are outlined in Supplementary Table S1. We selected three real-life DNA sequences obtained from the UCSC genome browser (Kent *et al.* 2002). The DNA sequences have lengths ranging from 1.6 ×${\;10}^{9}$ to 3.1 × $10^{9}$, and only contain the characters A, T, C, and G, with any instances of Ns within these DNA datasets converted to As.

Regarding the integer type used in the implementations, it is noted that while all test cases have lengths that can fit into unsigned 32-bit integer type, two DNA sequences (CHM13v2.0 and mouse, respectively) have lengths greater than what a signed 32-bit integer can represent. Since libsais and parDSS do not support unsigned 32-bit implementations, they use the 64-bit signed integer version for these two test cases and the 32-bit signed integer version for the others. Other algorithms consistently use signed 32-bit integers (see Supplementary Material S6 for details of the testing environment).

First, we tested the time utilization for a DNA test case, CHM13v2.0, which is the first complete sequence of a human genome. To enable direct comparisons, we measured the time required to construct suffix arrays, including the time for allocating memory for the suffix array.

**Figure 3.**
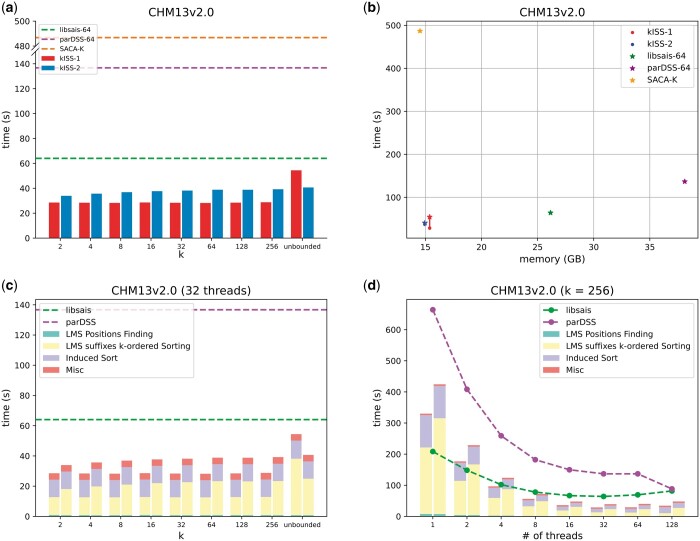
Performance for kISS in CHM13v2.0. (a) Runtime comparison of different algorithms. The *x*-axis represents different *k* values (with “unbounded” indicating unbounded *k*), and the *y*-axis shows the runtime for each algorithm. All algorithms, except for sequential SACAK, are executed with 32 threads. (b) Memory usage and runtime for various algorithms. The *x*-axis shows each algorithm's memory consumption, while the *y*-axis displays their runtime. Asterisks indicate unbounded values of *k*, and lines are drawn to represent trends across different *k* values. Again, all algorithms except for SACAK are run with 32 threads. (c) Runtime breakdown for each step of kISS across varying *k* values. The *x*-axis lists the different *k* values, and the *y*-axis illustrates the time each step takes. Each *k* value is represented by two bars, showing the runtime for kISS-1 (left) and kISS-2 (right). (d) Runtime breakdown for each step of kISS using different thread counts on the CHM13v2.0 test. The *x*-axis shows different thread numbers, while the *y*-axis indicates the runtime, with separate bars for kISS-1 (left) and kISS-2 (right). All tests are conducted with *k* = 256.

The time utilization of each algorithm for the CHM13v2.0 is graphically represented in Fig. 3a. For a detailed overview of outcomes from other tests, refer to Supplementary Fig. S1. At small *k* values, both strategies employed by kISS showcased superior performance compared to all other algorithms. Notably, for a commonly used *k* value of ranging from 32 to 256, frequently applied in short read searches, kISS-1 and kISS-2 exhibited speedups of approximately 2.22× and 1.62×, respectively, compared to libsais in CHM13v2.0.

It is noteworthy that kISS can outperform libsais even when *k* is unbounded. This advantage arises because the sorting algorithms utilized in kISS actively avoid the data dependency associated with the recursion required by the pSACAK+ algorithm, thereby enhancing parallelization.

Regarding the relative performance of the two strategies, kISS-1 displayed enhanced efficacy with *k* values below 256. In contrast, kISS-2 gained an edge with unbounded *k* in complex genome like CHM13v2.0, suggesting its better worst-case behavior. kISS-2 strikes a balance between the simplicity derived from both induced-copying and *k*-ordered idea, and the capacity to handle genomes with various properties.

Although the different *k* values have a limited impact on the performance of both strategies, the reasons for this are distinct. In kISS-1, the algorithm employs SIMD instructions to simultaneously compare a large number of characters, leading to stable performance when *k* values vary. On the other hand, kISS-2’s crucial step involves excluding singletons, effectively removing indices from a partial sort when their ranks are already determined. This results in a sharp decrease in the number of non-singletons with each round of doubling (see Supplementary Material S3 for more details).

Memory consumption for each test case is presented in Supplementary Tables S2 and S3. Generally, both algorithms use only slightly more additional memory than SACA-K, the most space-efficient state-of-the-art construction algorithm. Among the options, the 32-bit version of libsais ranks as the second most memory-efficient, outperforming all other algorithms except kISS.

Comparison of memory consumption and time utilization of each algorithm is presented in Fig. 3b and Supplementary Fig. S2. Both kISS-1 and kISS-2 exhibit superior performance in speed compared to all other algorithms. In addition, they only marginally increase memory usage compared to SACA-K and 32-bit version of libsais in large DNA sequences.

### 3.2 Parallelization for each step of kISS

Subsequently, we delved into a detailed analysis of the time consumed by each step. We categorized the runtime into the following four categories in kISS-1 and kISS-2: “LMS Positions finding,” “LMS suffixes *k*-ordered Sorting,” “Induced Sort,” and “Miscellaneous” (such as memory allocation). The results for the CHM13v2.0 test with different *k* values are presented in Fig. 3c, with additional results available in Supplementary Fig. S3. Figure 3c highlights that the “LMS suffixes k-ordered Sorting” and “Induced Sort” steps are the most time-intensive stages of the algorithm. Since the time spent on “Induced Sort” remains consistent across the same tests, the overall runtime is predominantly determined by the “LMS Suffixes *k*-Ordered Sorting” step. This step, which can be efficiently parallelized in kISS, demonstrated superior running times in real genome data, despite not having theoretically better time complexity.

The CHM13v2.0 test results for various threads are presented in Fig. 3d, while additional findings can be found in Supplementary Fig. S4. Figure 3d illustrates the notable scalability of the two algorithms. Specifically, when employing 2, 4, and 8 threads for the CHM13v2.0 test with *k *=* *256, kISS-1 achieved approximately 1.86×, 3.41×, and 5.86× speedups, respectively. In comparison, kISS-2 achieved approximately 1.85×, 3.43×, and 5.91× speedups under the same configurations. Compared to the recursive structure of libsais, kISS demonstrates better parallelization efficiency, making it more suitable for multi-core conditions with eight or more threads.

## 4 Discussion

kISS represents a sophisticated solution specifically engineered to optimize both time and space efficiency during the construction of *k*-ordered suffix arrays. This method leverages the ability to efficiently identify short seed sequences within large reference genomes. kISS facilitates the creation of *k*-ordered FM-indexes, as initially proposed by sBWT, by using *k*-ordered suffix arrays. kISS enables the effective integration of these *k*-ordered FM-indexes with the FMtree’s location function. This integration not only speeds up the construction of FM-indexes but also enhances their utilization without incurring additional computational costs.

The main innovation of kISS lies in its method of constructing *k*-ordered suffix arrays, inspired by induced sort principles. Unlike traditional methods that rely on reducing subproblems, kISS takes a direct approach by sorting all left-most S-type (LMS) suffixes. This enhances parallelism and takes advantage of the speed improvements inherent in *k*-ordered concepts.

Our study compared various suffix array construction algorithms and showed that kISS outperforms them significantly in terms of speed in large genomes with adequate multithreading. By analyzing the benefits of kISS LMS sorting strategies, we showed that Strategy 1 (kISS-1) offers a straightforward implementation and achieves the highest parallelism compared to recursive frameworks, making it suitable for multi-core setups. However, kISS-1 suffers from inferior worst-case time complexity, resulting in drastically increased time usage when there is an extremely high repetition in the prefixes of suffixes. Conversely, Strategy 2 (kISS-2) uses prefix-doubling to manage overall algorithm complexity and eliminate hard-to-parallelize steps. This approach is therefore less affected by the composition of reference genomes compared to kISS-1.

In general, both strategies prove the induced-copying framework of constructing suffix arrays to be efficient. In fact, the sorting method in between can be any existing suffix array construction algorithm, given the positions of suffixes (Strategy 1) or the compacted representation (Strategy 2) as input. This may inspire future works to also adopt this framework to improve our results.

A *k*-ordered FM-index inherently limits the maximum length of the query to *k*, as the search operations within the index cannot extend beyond the order of sorting. When the value sampling distance *vd* is also considered, this limitation tightens further to *k*−*vd *+* *1. In practical terms, if *k* is set too small, it restricts the index’s utility for matching longer seeds or patterns. Given these constraints, setting *k* to a very low value might improve construction speed but at the cost of severely limiting the utility of the index in practical scenarios. Based on empirical evidence and theoretical analyses, a *k* value of 256, with a *vd* of 4, appears to provide an optimal balance, accommodating sufficiently long patterns for effective seed matching while still enhancing performance and reducing the index size (about 5GB for CHM13v2.0).

## 5 Conclusions

Integrating *k*-ordered FM-indexes with kISS represents a pivotal advancement in genomic analysis, enhancing both efficiency and effectiveness. The innovative *k*-ordered approach employed by kISS, especially through the use of two distinct sorting strategies, contributes significantly to its superior performance relative to traditional suffix array construction methods. By leveraging these techniques, kISS demonstrates superior speed and memory utilization, particularly within the context of large genomes. The highly parallel nature of kISS ensures competitive performance even when *k* is unbounded, which means kISS can also efficiently construct the traditional suffix array. kISS not only advances the computational handling of genomic data but also integrates seamlessly into existing bioinformatics workflows, promising substantial improvements in the processing of increasingly complex genomic libraries.

## Supplementary Material

btae409_Supplementary_Data

## Data Availability

All the genome assemblies (CHM13v2.0, GRCm39, GRCz11) were downloaded from the National Center for Biotechnology Information (NCBI). The links to these genome assemblies are listed below. CHM13v2.0: https://www.ncbi.nlm.nih.gov/datasets/genome/GCF_009914755.1/. GRCm39: https://www.ncbi.nlm.nih.gov/datasets/genome/GCF_000001635.27/. GRCz11: https://www.ncbi.nlm.nih.gov/datasets/genome/GCF_000002035.6/.
