# Supplementary material for: Efficient construction and utilization of k-ordered FM-indexes with kISS for ultra-fast read mapping in large genomes
Source: Bioinformatics. 2024 Jun 19;40(7):btae409. doi: 10.1093/bioinformatics/btae409 (PMC11269432; doi:10.1093/bioinformatics/btae409)
Supplement: btae409_Supplementary_Data [file btae409_supplementary_data.zip › supplementary.docx]

# Supplementary Materials

**Efficient Construction and Utilization of k-Ordered FM-indexes**

**with kISS for Ultra-Fast Read Mapping in Large Genomes**

Zheng-Dao Yang^1,†^, Hsuan-Yu Kuo^1,†^, Po-Wei Hsieh^1^ and Jui-Hung Hung^1,*^

^1^Department of Computer Science, National Yang Ming Chiao Tung University, Hsinchu, Taiwan

^†^Equally contributing authors

^*^To whom correspondence should be addressed:

Jui-Hung Hung, Department of Computer Science, National Chiao Tung University, No. 1001, Daxue Rd. East Dist., Hsinchu City 300093, Taiwan

Phone: +886-3-5712121 ext 59267; E-mail: [jhh@cs.nycu.edu.tw](mailto:jhh@cs.nycu.edu.tw) or [juihunghung@gmail.com](mailto:juihunghung@gmail.com)

# S-1 Suffix Array Algorithms

***Suffix Array.*** The suffix array of a string *S*[0..*n*−1] with length *n* ≥ 1 arranges its suffixes *S*[*i*..*n*−1] in ascending lexicographical order. Typically, it is assumed that *S* concludes with the smallest letter in the string, occurring only once. This character (or suffix) is often referred to as the sentinel. For instance, in the string *S* = “ATACT$”, the suffix array would be [5,2,0,3,4,1]. Here, the sentinel *S*[5..5] = “$” represents the smallest suffix, followed by *S*[2..5] = “ACT$,” and so forth. The character ranking is $ < A < C < G < T in this example. The application of suffix arrays includes full-text indices and data compression. (Puglisi, Smyth et al. 2007)

In this work, we focus on two major categories of suffix array construction algorithms: induced-sorting-based algorithms and prefix-doubling algorithms.

***Induced-sorting-based algorithms (including recursive algorithms and Induced Copying Algorithms).*** Algorithms from this category heavily rely on the concept of induced sorting. The principle behind induced sorting is that the order of a selected subset of suffixes can be used to reconstruct the complete order of all suffixes. In most induced-sorting-based algorithms, the induced sorting step can be efficiently performed, typically in time linear to the input size.

For example, the widely used SA-IS algorithm (Nong, Zhang et al. 2009) leverages the fact that once the order of suffixes from the LMS (Leftmost S-type) category (or LMS suffixes) is known, the entire suffix array can be induced in linear time. The LMS suffixes are determined as follows: First, the algorithm determines whether each suffix belongs to an S-type or L-type category. Specifically, a suffix *S*[*i*..*n*−1] is termed S- or L-type based on whether *S*[*i*..*n*−1] < *S*[*i*+1..*n*−1] or *S*[*i*..*n*−1] > *S*[*i*+1..*n*−1] in lexicographical order, respectively. (Note that *S*[*i*..*n*−1]=*S*[*i*+1..*n*−1] can never hold because they have different lengths). Conventionally, the sentinel is defined to be S-type. Then, the LMS suffixes are the suffixes *S*[*i*..*n*−1] where *S*[*i*..*n*−1] is S-type and *S*[*i*−1..*n*−1] is L-type (1 ≤ *i* ≤ *n*−1). All *i* satisfying this constraint are called the LMS characters. In this context, the list of LMS suffixes can be found in a single linear scan. The final induce sort can also be executed in linear time as shown in ***Algorithm S-1***.

To efficiently sort the LMS suffixes, the SA-IS algorithm adopts a recursive framework and reduces it to a subproblem. The algorithm retrieves all substrings between two consecutive LMS characters, which are referred to as the LMS substrings. Subsequently, it sorts all the LMS substrings in lexicographical order, replaces each substring with its rank, and reorders the ranks in the original positional order. Finally, the reordered rank is regarded as the input of the subproblem. This technique is commonly known as substring naming.

In summary, the steps of the SA-IS algorithm in each recursive step include the discovery of LMS suffixes, substring naming, reduction to a subproblem, and induced sort. Note that all these steps in this framework can be done in linear time; the key is that substring naming can be solved in O(*n*) after another induced sorting, which sorts all LMS substrings and is the same algorithm as ***Algorithm S-1*** except that the LMS suffixes can be in any order. Since the number of LMS suffixes is at most half of the reference size, the time complexity for the whole algorithm T(*n*) can be written as the formula T(*n*)=O(*n*)+T(*n*/2). Thus, T(*n*)=O(*n*) according to the Master's theorem. Several successors are inspired by this framework, including SACAK (Nong 2013) and pSACAK+ (Xie, Lao et al. 2020). This paradigm remains the fastest suffix sorting option in the current state-of-the-art.

***Prefix-doubling Algorithms.*** Prefix-doubling algorithms extend the length of the prefix of all suffixes in a partial sort, where suffixes are ordered based on the first *h* characters of each suffix, with *h* being a predefined integer. Specifically, if *SA_h_*[*i*] denotes the *i*-th ranked suffix considering only the first *h* characters, and *ISA_h_*[i] denotes the rank of the suffix *S*[*i*..*n*] (it is in fact the pseudorank, i.e. *ISA*[*i*] is the number of suffixes that has strictly smaller *h*-mer prefix than the *i*-th suffix’s *h*-mer prefix), then a sort using the key (*ISA_h_*[*i*], *ISA_h_*[*i* + *h*]) is in fact *SA*_2_*_h_*[*i*]. The sorting process is known as doubling. The whole algorithm involves ordering suffixes by their first character and performing doubling log_2_(*n*) times, updating *ISA_h_* at each step, to obtain the correct suffix array. The algorithmic workflow of prefix doubling can be seen in ***Algorithm S-2***.

The bottleneck of prefix-doubling algorithms lies in the extensive usage of integer pair sorting. Parallel range algorithms from PBBS (Shun, Blelloch et al. 2012) offer efficient implementations for parallelized prefix doubling, reducing the number of integer pairs involved in sorting. One such technique is the segmented sort, where *SA_h_*[*i*] is ordered by the key *ISA_h_*[*i*], and suffixes with the same *ISA_h_*[*i*] form continuous segments in the array *SA_h_*. Each thread is responsible for sorting segments within a block by key *ISA_h_*[*i* + *h*], enabling parallelism. When a segment spans the boundary between two blocks, two threads sort a part of the segment, and a single thread merges the two sorted lists. Adopting a merge-sort-like procedure, one can also deal with cases when a segment spans across multiple segments.

Another technique involves recognizing that if a segment contains only a single element (it is called a singleton), its final rank is known, and the singleton can be removed from further doubling steps. These innovations improve the efficiency of prefix-doubling algorithms, making them more practical for large-scale applications.

# S-2 Construction of FM-indexes and FMtree

The FM-index, a data structure for efficient pattern searching, consists of *SA*, BWT(*S*), *C*, and *OCC*, related to suffix arrays. *SA* is the suffix array of *S*, BWT(*S*) is the Burrows-Wheller transform relying on the suffix array, *C*[*c*] is the count of occurrences of characters from 1 to *c* – 1 in *S*, and *OCC*[*i*][*c*] is the count of occurrences of *c* in the prefix BWT(*S*)[0, *i* - 1]. In the *k*-ordered version, the suffix array is substituted with a k-ordered suffix array *kSA*. Subscript sampling distance (*sd*) and value sampling distance (*vd*) are defined for *OCC* and *SA* (or *kSA*) respectively, where only entries with *i* mod *sd* = 0 for *OCC* and *SA*[*i*] mod *vd* = 0 for *SA* (or *kSA*) are saved.

The LF (Last-to-First) function plays a crucial role in count and locate operations. It is defined as *C*[*c*] + *OCC*[*l*][*c*] (or LF(*l*, *c*)). LF-mapping(*l*) refers to the LF function with *c* = BWT(*S*)[*l*]. (Ferragina and Manzini 2000)

For value-sampled suffix arrays, locating patterns can be challenging. However, a key observation states that for two positions *SA*[*i*] and *SA*[*j*] with BWT(*S*)[*i*] = BWT(*S*)[*j*] = *c*, if no character *c* exists between them, then LF-mapping(*i*) = LF-mapping(*j*) - 1 must hold. Thus, let *SA*[*sp*, *ep*] be all positions with pattern *P*, then *SA*[LF-mapping(*sp_c_*), LF-mapping(*ep_c_*)] are all positions with pattern *cP*, where *sp_c_* and *ep_c_* are the first occurrence and last occurrence of *c* in BWT(*S*)[*sp*, *ep*]. Since LF-mapping(*sp_c_*) = LF(*sp*, *c*) and LF-mapping(*ep_c_*) = LF(*ep*, *c*) must hold, the positons with pattern *cP* can be rewritten as *SA*[LF(*sp*, *c*), LF(*ep*, *c*)]. Applying this observation recursively enables retrieval of segments with pattern *sP*, where *s* can be any prefix. Increasingly enumerating all possible prefixes of length up to *vd* guarantees that all suffixes of *SA*[*sp*, *ep*] are properly obtained. This observation forms the basis of the sBWT algorithm, which efficiently retrieves positions with a specific pattern.

Moreover, the FMtree optimization further enhances the efficiency of locating patterns. It organizes strings into a complete |*Σ*|-ary tree (where |*Σ*| represents the alphabet size), with each node containing a string that precedes its parent's string by one character. This hierarchical organization enables efficient breadth-first search in the tree to find the range for a given pattern *P*, followed by block-by-block retrieval of occurrence positions in the sampled suffix array.

| **Algorithm S-1** Induced Sort |
| --- |
| **Input** Reference *S*, bucket array *BA*, list of LMS suffixes *LMS*  **Output** The sorted suffix array *SA*   1. Count the occurrences of each character, and define the range (or bucket) for each starting character in the suffix array 2. Initialize *BA* to be the indices of the buckets’ head 3. Initialize *SA* to be empty array of size *S*.size() 4. Put the LMS suffixes into the end of the bucket according to their first characters, in the order of their appearance in each bucket 5. **for** *i* = 0 to *S*.size() – 1 do 6. **if** *SA*[*i*] is not empty and *j* = *SA*[*i*] – 1 ≥ 0 and suffix *j* is L-type then 7. *SA*[*BA*[*S*[*j*]]++] = *j* 8. **end** **if** 9. **end for** 10. Initialize *BA* to be the indices of the buckets’ tail 11. **for** *i* = *S*.size() - 1 to 0 do 12. **if** *SA*[*i*] is not empty and *j* = *SA*[*i*] – 1 ≥ 0 and suffix *j* is S-type then 13. *SA*[--*BA*[*S*[*j*]]] = *j* 14. **end** **if** 15. **end** **for** |

| **Algorithm S-2** The Prefix-doubling Algorithm |
| --- |
| **Input** Reference *S*  **Output** The suffix array *SA*   1. Initialize *SA*[*i*] = *i* for *i* from 0 to *SA*.size() – 1 2. Initialize *ISA* be empty array of length *SA*.size() 3. Sort *SA* by key *S*[*i*] 4. *len* = 1 5. **while** *len* ≤ *S*.size() **do** 6. Sort *SA* by key (*ISA_h_*[*i*], *ISA_h_*[*i* + *h*]) 7. Update *ISA*[*i*] = the pseudorank of the *i*-th suffix in SA 8. *len* = 2 × *len* 9. **end** **while** |

# S-3 Implementation Details of kISS

***kISS software.*** The kISS software comprises three main functionalities: suffix array construction, FM-index construction, and pattern searching within the FM-index. In suffix array construction, users can choose between two algorithms (kISS-1 and kISS-2) and specify the prefix sorting length, denoted as *k*. The latter two functionalities implement the ordinary FM-index construction and the locate function, respectively, utilizing the FMtree algorithm.

The software accepts DNA sequences in fasta format as input. DNA sequences are encoded as character strings ranging from 0 to 3, corresponding to the nucleotides A, C, G, and T, respectively. Any other characters in the input are converted to 4, which is then mapped to 0 in all experiments.

Implemented purely in modern C++, the software leverages the OpenMP library for parallelism and integrates the Intel oneAPI Threading Building Blocks (oneTBB) library for execution policies. To enhance efficiency, a custom memory allocator is defined to align std::vector on a 64-byte boundary.

***LMS Positions Finding, Put LMS suffixes and Induced Sorting.*** Our implementation closely follows the approach in the libsais (v.2.8.1) library, which is known for its efficient pSACAK+ algorithm. We have incorporated optimizations from libsais and verified that our implementations achieve similar efficiency.

***Parallel k-ordered LMS suffix sort in kISS-1.*** The algorithm leverages SIMD-optimized suffix sorting. We first perform a 2-bit packing of the input, encoding each character into 2 bits, allowing for SIMD instructions. Next, the suffixes are radix-sorted based on their first 10-mers, resulting in 4^10^ = 1048576 buckets. Subsequently, multiple threads use std::sort with SIMD intrinsics to sort the buckets, comparing 125 characters at a time for easier implementation. The SIMD intrinsics in our implementations are AVX2 instructions (for easy handling and comparison of 256-bit data) and bitwise operations.

***Parallel k-ordered LMS suffix sort in kISS-2.*** Our implementation is based on the parallel range algorithm. The algorithm breakdown is as follows (Refer to ***Section S-1*** for definitions used):

The initial step involves the compacted representation of the reference. Given the reference string *S*, the LMS suffix positions, and the character set size |*Σ*|, the algorithm computes the parameter *l* by determining the number of bits required to represent one character divided by the bits needed for encoding elements in the character set. Subsequently, it encodes each LMS substring as integers and concatenates them in their original order to form a new reference, *cS*. Additionally, a Boolean array, *valid_pos*, is computed to indicate if the head of each *l*-mer originates from an LMS suffix, facilitating the extraction of the ordered LMS suffixes. We set *l*=16 in all the experiments.

Next is the main parallel range algorithm. Initially, it sorts the suffixes based on their first character. During implementation, both the suffix array (*SA*) and the pseudorank array (*ISA*) are calculated. Consequently, a list of segments with equal ranks (or segments for short) is obtained. To aid implementation, the head of each segment is marked in a Boolean array. In this fashion, the segments can be represented as continuous subsequences by adjacent heads of segments. Each doubling step comprises four stages: splitting, merging, updating *ISA*, and compacting.

- The splitting step divides the suffix array into blocks and sorts each segment within blocks by key *ISA_h_*[*i* + *h*] using std::sort in parallel.
- The merging step merges the sorted segments using a merge-sort-like process. Initially, there are *num_threads* blocks, with *num_threads* representing the number of employed threads. Each thread is responsible for merging the segment spanning across adjacent blocks, and every two blocks are amalgamated. This process continues until the number of blocks reduces to 1.
- The update *ISA* step updates the *ISA* array so that it is indeed *ISA_2h_*. It employs a linear algorithm to do so. The Boolean array storing the head of each segment is also updated in this step.
- The compacting step excludes segments with only one element (or singletons) and compactifies the remaining segments.

Following the parallel range algorithm, distinct ranks are assigned to elements in the remaining segments, resulting in the ordered suffix array of the compacted representation. Finally, using the previously calculated *valid_pos* to identify the true LMS suffixes, the ordered LMS suffixes are extracted. It's worth noting that, to conserve memory consumption, the finding LMS algorithm is rerun.

# S-4 The rationale for the encoding step

Encoding strings into arrays of integers facilitates fast comparison between two strings. By treating each *l*-mer as a base-|*Σ*| number, it decreases the number of comparisons of two *l*-mers from *l* to 1. Consequently, comparing two strings *S*_1_ and *S*_2_ can be achieved in roughly max(|*S*_1_|, |*S*_2_))/*l* comparisons. Given the objective of sorting all the LMS suffixes, we opt to encode each LMS substring to encompass all LMS indices.

When breaking down LMS substrings into *l*-mers, if the last substring has fewer than *l*-mers, the prefixes of the following LMS substrings are concatenated. This ensures the proper order of the suffixes. Let's consider comparing two LMS suffixes, *S*[*i*_1_..*n*-1] and S[*i*_2_..*n*-1], where the LMS substring *S*[*i*_1_, *j*_1_] is encoded into an array *a*, and the LMS substring *S*[*i*_2_, *j*_2_] is encoded into an array *b*. Without loss of generality, assume |*a|* ≤ |*b|*, where |*a|* and |*b|* are the lengths of *a* and *b* respectively. Here are the scenarios:

***Case 1***. If *a* ≠ *b* and *a* is not a proper prefix of *b*, then the prefixes of length |*a|* × *l* are distinct. Thus, a comparison of the two arrays yields the correct outcome.

***Case 2***. If *a* ≠ *b* and *a* is a proper prefix of *b*, the comparison involves *l*-mers at positions *j*_1_ and *i*_2_ + *a* × *l*. This compares substrings S[*i*_3_.. *j*_3_] and S[*i*_2_ + *a* × *l*.. *j*_2_], where S[*i*_3_.. *j*_3_] begins at *i*_3_ = *j*_1_. Notably, S[*i*_2_ + *a* × *l*] is L-type (S[*i*_2_ + (*j*_1_ - *i*_1_) - 1] = S[*j*_1_ - 1] > S[*j*_1_] = S[*i*_2_ + (*j*_1_ - *i*_1_)], *i*_2_ + (*j*_1_ - *i*_1_) – 1 < *i*_2_ + *a* × *l* and S[*i*_2_ + *a* × *l*] is in the same LMS substring as S[*i*_2_ + (*j*_1_ - *i*_1_) - 1], which is L-type) and S[*i*_3_] is S-type, either S[*i*_2_ + *a* × *l*] ≠ S[*i*_3_], or the first characters diverging from the starting characters differ in the two substrings. By comparing the initial characters where differences occur (up to the minimum of their lengths), the correct outcome is inferred.

***Case 3.*** If the encoded integer arrays are identical, it signifies that the two LMS substrings starting at *i*_1_ and *i*_2_ are the same. The correct result is deduced by further comparing the integer arrays associated with the LMS substrings starting at *j*_1_ and *j*_2_.

In either case, incorporating the subsequent LMS substrings consistently produces the correct result.

# S-5 The correctness proof using FM-tree with *k*-ordered FM-indexes

To begin with, we define a *h*-group to be a maximal group of suffixes with equal prefixes of length *h*. Note that it always spans continuously in ordinary suffix arrays and *k*-ordered suffix arrays with *h* ≤ *k*.

***Theorem 1***. If a *k*-group locates at the range [*sp*, *ep*] of the suffix array, the LF function on *sp* produces identical outcome, irrespective of whether *k*-ordered FM-indexes or conventional FM-indexes are employed.

***Proof***. It's important to note that while the order of suffixes may differ within each *k*-group in *k*-ordered suffix array when compared to its counterpart, the ordering of the *k*-groups remains unaltered. Therefore, for a specific *k*-group [*sp*, *ep*] in *k*-ordered ones, the set of *k*-groups preceding *sp* is identical to that of the conventional suffix arrays, yielding the collection of suffixes preceding *i = sp* remains. Consequently, the value *OCC*[*i*][*c*] in the LF function, which is the number of *j* such that *j* < *i* and *S*[*kSA*[*j*] - 1] = *c*, retains its consistency for *i* = *sp*. *C*[c] is apparently constant, thereby ensuring a uniform value of LF(*i*, *c*) for *i* = *sp*.

An immediate implication arising from ***Theorem 1*** is that, if [*sp*, *ep*] defines some *i*-group and *i* ≤ *k*, then LF operations on *sp* and *ep*+1 give accurate results. Any *i*-group is the concatenation of multiple *k*-groups, so *sp* is the head of the first *k*-group whereas *ep* + 1 is the head of the subsequent *k*-group, rendering ***Theorem 1*** applicable. This implication leads to the subsequent theorem:

***Theorem 2***. With the integration of *k*-ordered FM-indexes, the FMtree gives identical *locate* results to those obtained using conventional FM-indexes when the length of the query does not exceed *k – vd +* 1.

***Proof***. FMtree starts with the *count* operation on the given query *P*. In the *i*-th iteration of the count function, the LF operations are only applied on *sp* end *ep* + 1, where [*sp*, *ep*] defines an *i* – 1-group; the above implication is therefore applicable. Traversing the FMtrees, we are applying LF operations on *sp* end *ep* + 1 and [*sp*, *ep*] is the range prefixed by *sP*. Since each *s* has length at most *vd* – 1 (the height of the tree is *vd*, so |*s*| from non-leaf node is at most *vd* - 1), from |*P*| ≤ *k* - *vd* +1, we have |*P*| + |*s*| ≤ (*k* – *vd* + 1) + (*vd* - 1) = *k*, rendering ***Theorem 1*** applicable. Therefore, the ranges explored by FMtrees remain the same.

Furthermore, within *k*-ordered suffix arrays, each sampled position remains in the same *k*-group as in conventional suffix arrays. Given that each range explored by FMtree comprises the amalgamation of multiple *k*-groups, the set of sampled positions within that range remains unaltered. Consequently, the collection of indexes explored by FMtree remains unchanged when the query length does not exceed *k – vd + 1*.

# S-6 Experimental Details

The benchmark code is located in the experiment folder of the repository. To ensure a fair comparison between suffix array construction algorithms, we define runtime as the time taken to construct the suffix array, including the time for memory allocation. Each valid configuration of algorithms, number of threads, and *k* is tested three times, and the average of these three runtimes is taken. To minimize the impact of running on different cores, we specify the cores for multithreaded algorithms using the taskset command in Linux. Specifically, when the number of threads is less than 128, it utilizes every other core. For 128 threads, it utilizes all available cores.

# S-7 Supplementary experimental results

**Table S1.**The testing datasets for the experiments. *n* is the length of the reference, while *|Σ|* is the size of the character set.

**Table 1.**The testing datasets for this experiment. *n* is the length of the reference, while *|Σ|* is the size of the character set.

| Dataset | n | \|Σ\| | Description |
| --- | --- | --- | --- |
| CHM13v2.0 | 3117292070 | 4 | Human sample from CHM13htert cell line from Homo sapiens. The first complete sequence of a human genome. |
| mouse | 2728222451 | 4 | The Jun. 2020 (GRCm39/mm39) assembly of the mouse genome. |
| zebrafish | 1679203469 | 4 | The May 2017 (GRCz11/danRer11) assembly of the zebrafish genome. This dataset shares much more repeats between the suffixes. |

**Table S2.** The memory consumption for each dataset, measured in Gibibytes (GiB), can be calculated through the ru_maxrss variable, which captures the maximum resident set size using the getrusage POSIX function.

| Dataset | kISS-1 | kISS-2 | libsais (64-bit) | libsais (32-bit) | parDSS (64-bit) | parDSS (32-bit) | SACA-K |
| --- | --- | --- | --- | --- | --- | --- | --- |
| CHM13v2.0 | 15.381 | 14.955 | 26.151 | N/A | 38.110 | N/A | 14.520 |
| mouse | 13.478 | 13.110 | 22.889 | N/A | 33.880 | N/A | 12.708 |
| zebrafish | 8.349 | 8.125 | 14.082 | 7.829 | 20.489 | 12.619 | 7.823 |

**Table** **S3.**The memory consumption for each dataset, in bytes per character. The results for kISS-1 and kISS-2 are for *k*=256.

| Dataset | kISS-1 | kISS-2 | libsais (64-bit) | libsais (32-bit) | parDSS (64-bit) | parDSS (32-bit) | SACA-K |
| --- | --- | --- | --- | --- | --- | --- | --- |
| CHM13v2.0 | 5.298 | 5.151 | 9.007 | N/A | 13.126 | N/A | 5.001 |
| mouse | 5.304 | 5.159 | 9.008 | N/A | 13.334 | N/A | 5.001 |
| zebrafish | 5.339 | 5.195 | 9.004 | 5.006 | 13.101 | 8.069 | 5.002 |

**Table S4.**The length, number of LMS suffixes and length of the reduced reference in each test.

**Table 1.**The testing datasets for this experiment. *n* is the length of the reference, while *|Σ|* is the size of the character set.

| Dataset | Length | # of LMS suffixes | Length of the compacted reference in kISS-2 (with *l*=16) |
| --- | --- | --- | --- |
| CHM13v2.0 | 3117292070 | 868860623 | 869312888 |
| mouse | 2728222451 | 761201338 | 765827418 |
| zebrafish | 1679203469 | 477089626 | 477521561 |


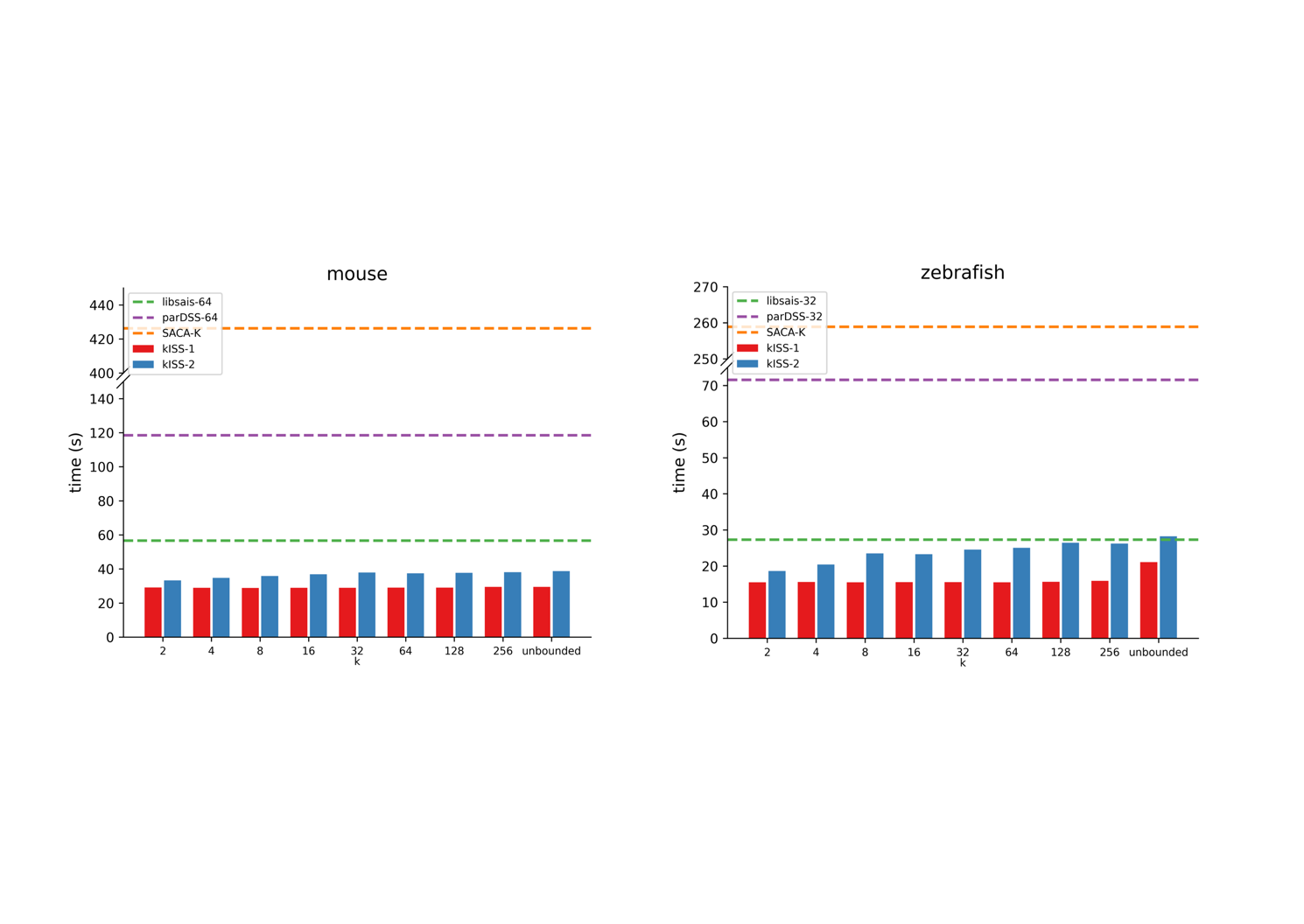


**Figure S1.** Runtime for different algorithms on various tests. The x-axis displays the result for different ks (unbounded stands for unbounded k), while the y-axis represents the running time for each algorithm. Algorithms other than SACAK (which is sequential) are run with 32 threads.


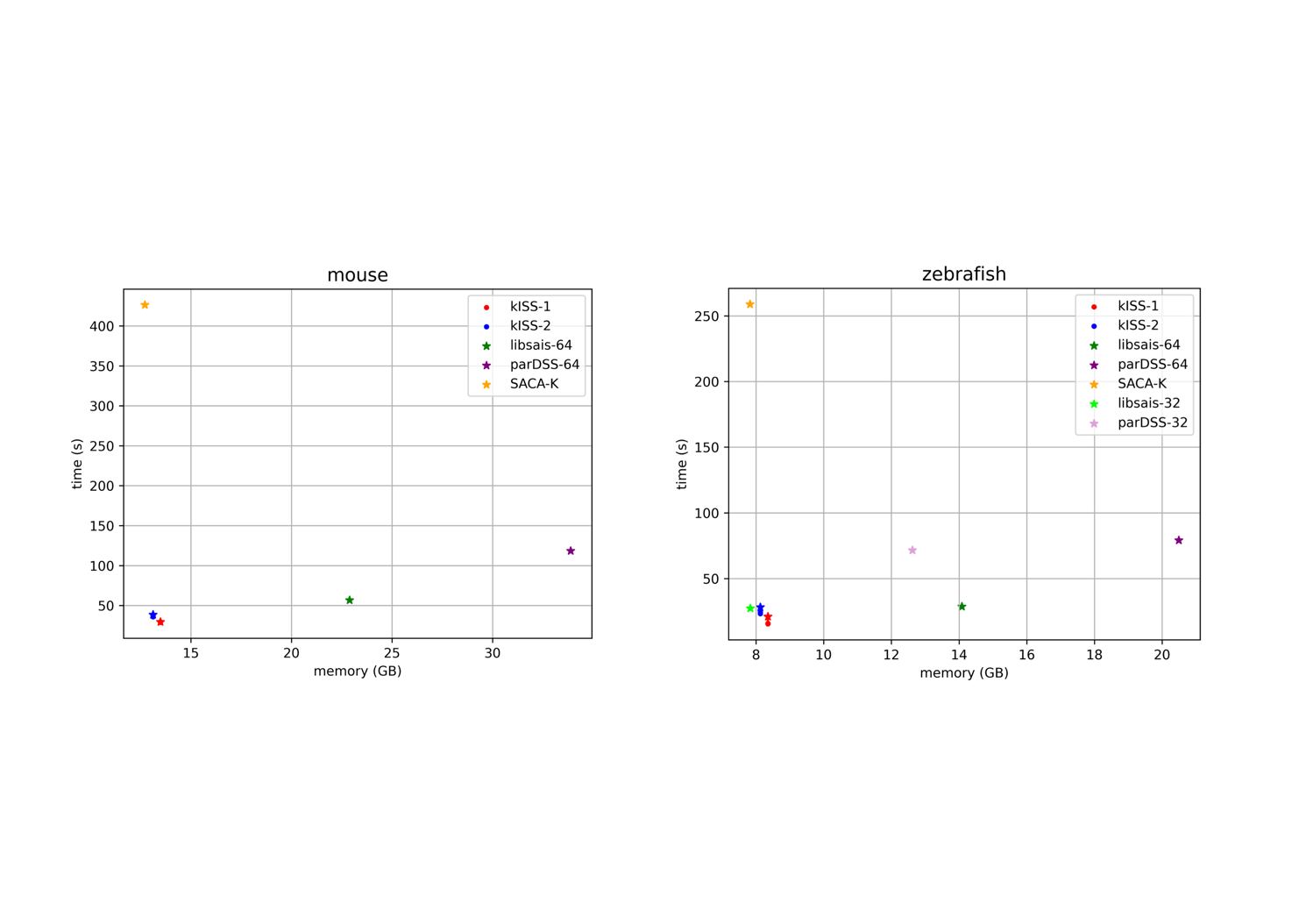

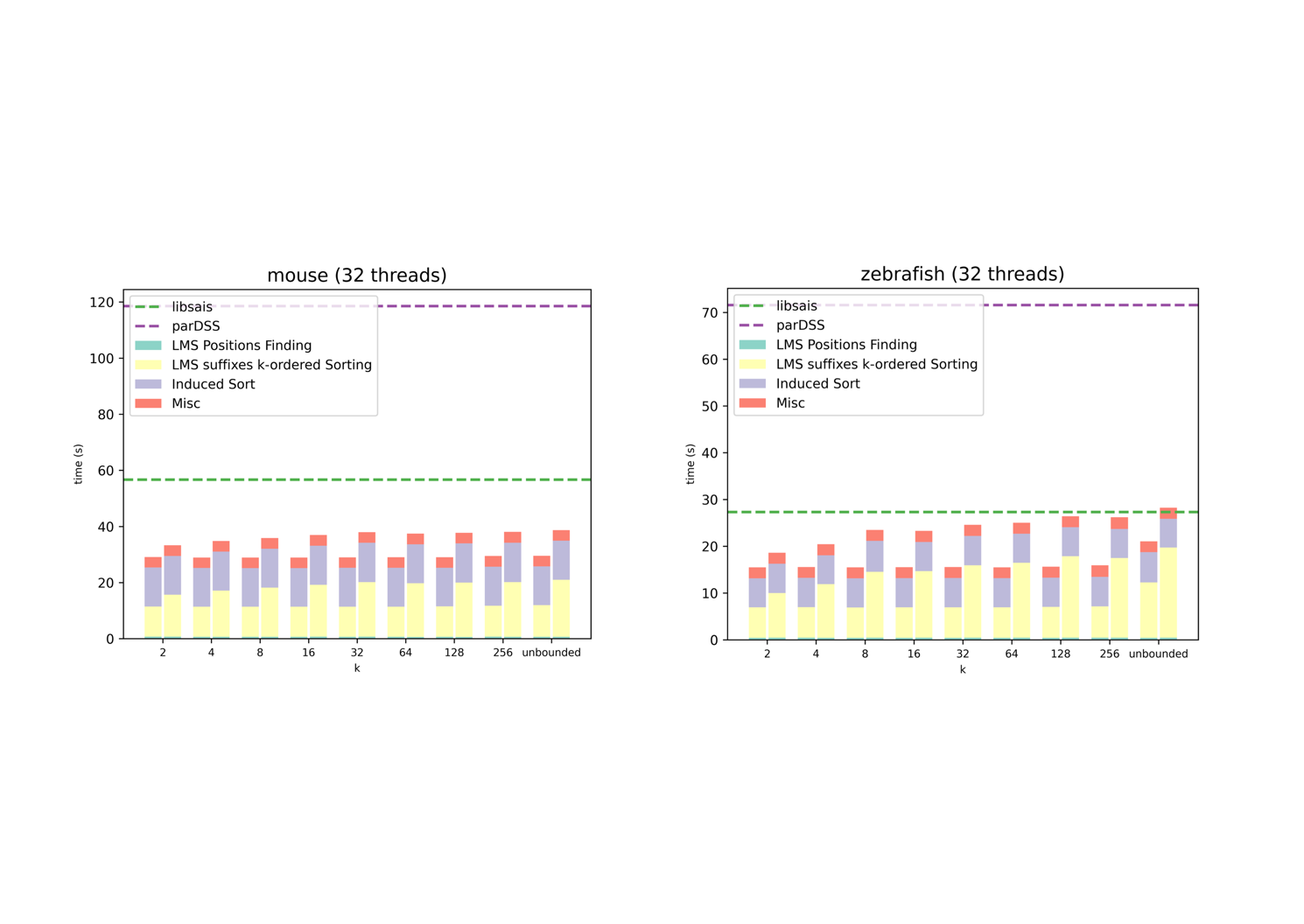


**Figure S2.** Memory usage and time for different algorithms on various tests. The x-axis represents the memory usage of each algorithm, while the y-axis represents the time usage of each algorithm. Asterisks denote unbounded values of k, while lines represent straight lines drawn for different values of k.



**Figure S3.** Time usage for each step of kISS with varying k values on various tests. The x-axis denotes different k values, while the y-axis illustrates the running time. Each k value is represented by two bars, indicating the running time for kISS-1 (on the left) and kISS-2 (on the right), respectively. Algorithms are run with 32 threads.


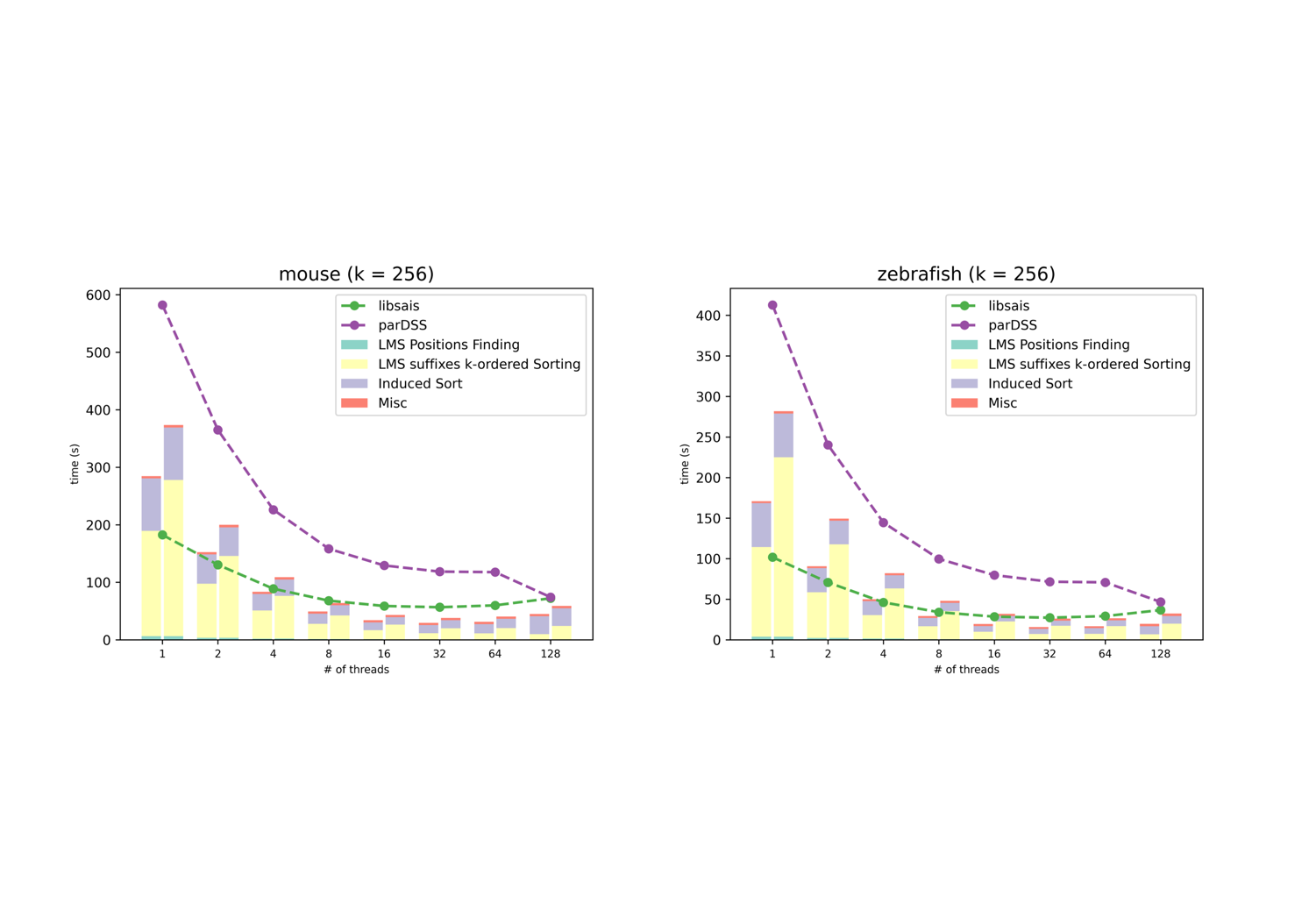


**Figure S4.** Time usage for each step of kISS with different number of threads on various tests. The x-axis denotes different numbers of threads, while the y-axis represents the running time. The two bars indicate the running time for kISS-1 (on the left) and kISS-2 (on the right), respectively. Algorithms are run with k = 256.

# References

Ferragina, P. and G. Manzini (2000). Opportunistic data structures with applications. Proceedings 41st annual symposium on foundations of computer science, IEEE.

Nong, G. (2013). "Practical linear-time O(1)-workspace suffix sorting for constant alphabets." ACM Transactions on Information Systems **31**(3): 1-15.

Nong, G., S. Zhang and W. H. Chan (2009). Linear Suffix Array Construction by Almost Pure Induced-Sorting. 2009 Data Compression Conference**:** 193-202.

Puglisi, S. J., W. F. Smyth and A. H. Turpin (2007). "A taxonomy of suffix array construction algorithms." ACM Computing Surveys **39**(2).

Shun, J., G. E. Blelloch, J. T. Fineman, P. B. Gibbons, A. Kyrola, H. V. Simhadri and K. Tangwongsan (2012). Brief announcement: the problem based benchmark suite. Proceedings of the twenty-fourth annual ACM symposium on Parallelism in algorithms and architectures. Pittsburgh, Pennsylvania, USA, Association for Computing Machinery**:** 68–70.

Xie, J. Y., B. Lao and G. Nong (2020). In-Place Suffix Sorting on a Multicore Computer with Better Design. Parallel Architectures, Algorithms and Programming**:** 331-342.
