# Supplementary figures and images for: Efficient construction and utilization of k-ordered FM-indexes with kISS for ultra-fast read mapping in large genomes
Source: Bioinformatics. 2024 Jun 19;40(7):btae409. doi: 10.1093/bioinformatics/btae409 (PMC11269432; doi:10.1093/bioinformatics/btae409)

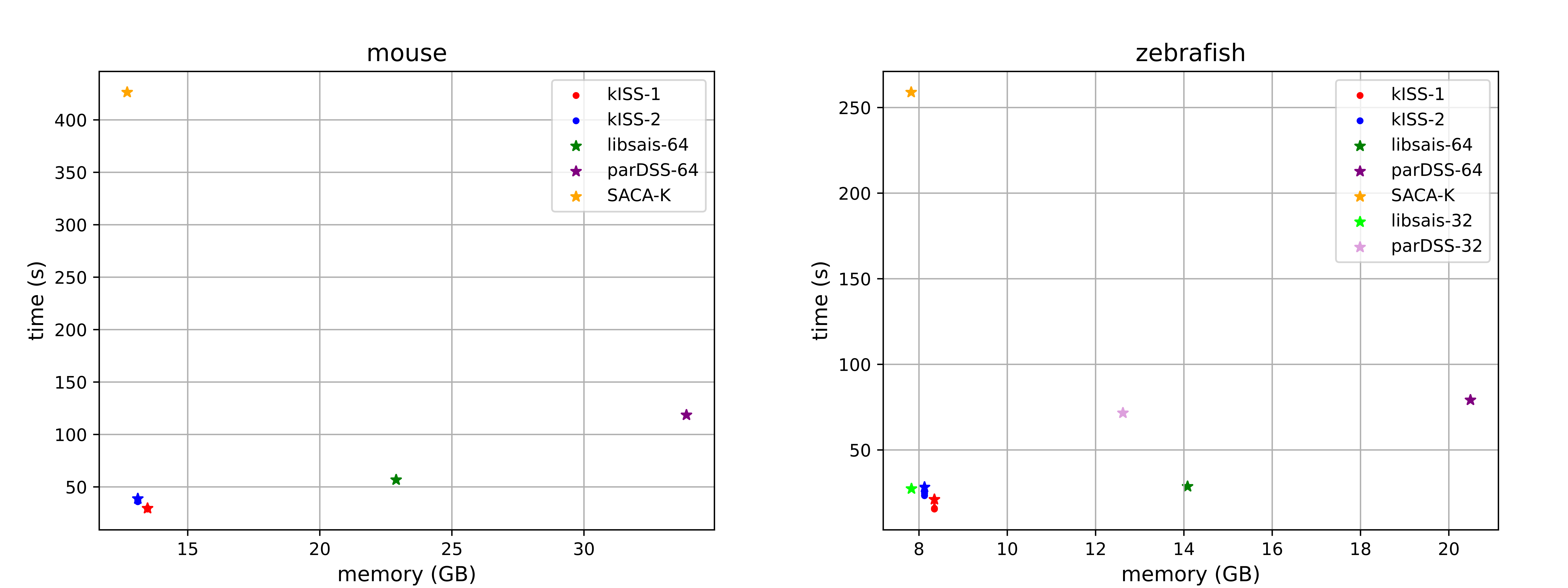

Supplement: btae409_Supplementary_Data [file btae409_supplementary_data.zip › figure_S2.png]

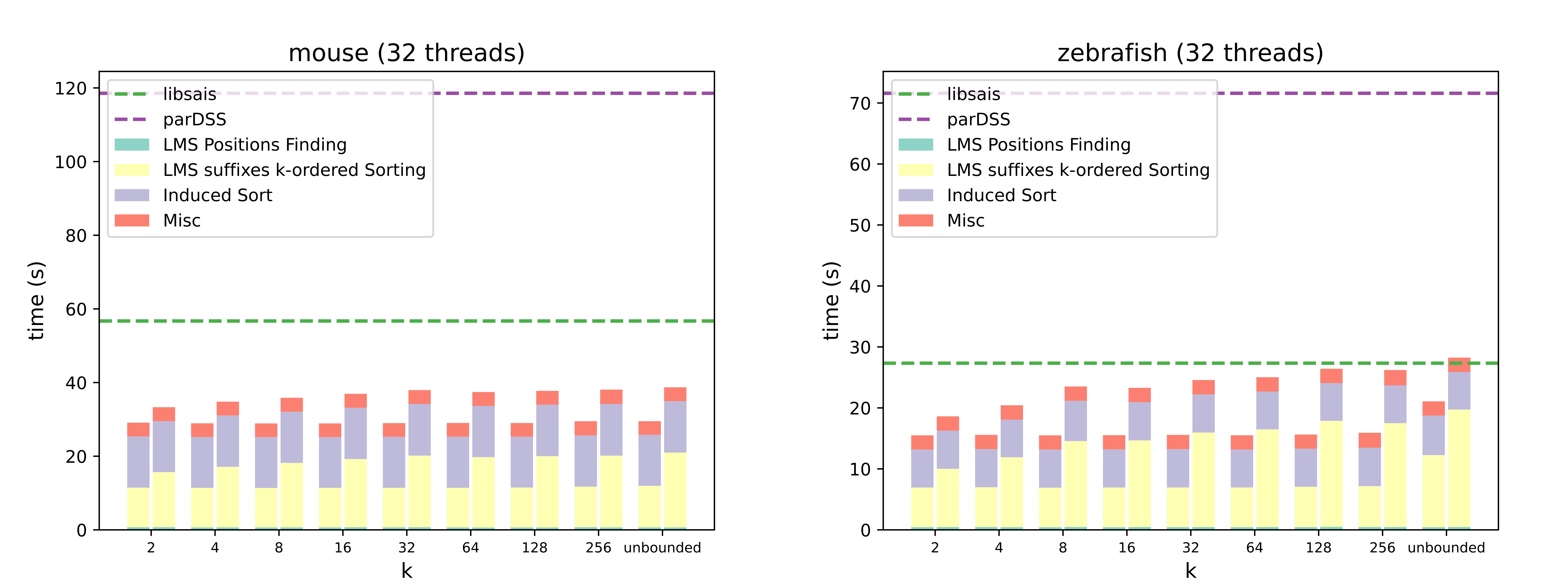

Supplement: btae409_Supplementary_Data [file btae409_supplementary_data.zip › figure_S3.png]

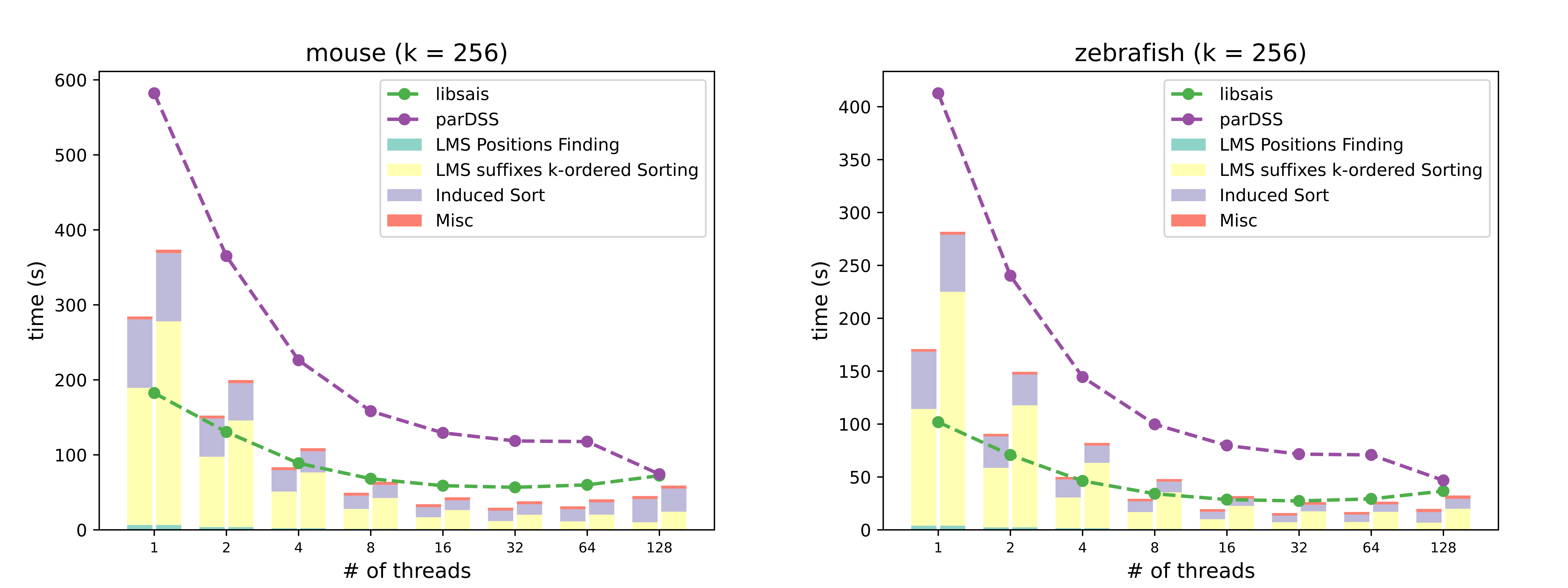

Supplement: btae409_Supplementary_Data [file btae409_supplementary_data.zip › figure_S4.png]

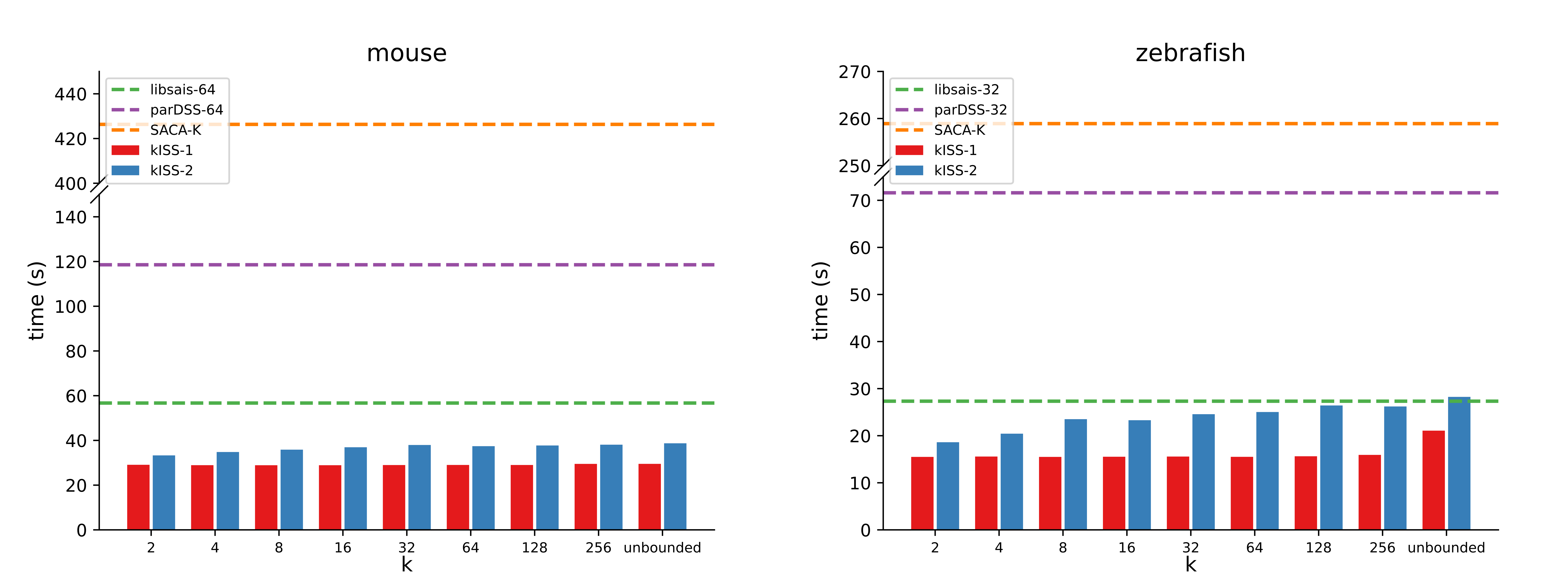

Supplement: btae409_Supplementary_Data [file btae409_supplementary_data.zip › figure_S1.png]
